# Author Correction: In situ cryo-ET defines the ultrastructure of ER exit sites in human cells

**DOI:** 10.1038/s41556-026-02035-2

**Published:** 2026-07-08

**Authors:** Katie W. Downes, Julia R. Flood, Andrea Nans, Sander E. Van der Verren, Anjon Audhya, Giulia Zanetti

**Affiliations:** 1 Institute of Structural and Molecular Biology, University College London, London, UK; 2 The Francis Crick Institute, London, UK; 3 Department of Biomolecular Chemistry, University of Wisconsin School of Medicine and Public Health, Madison, WI USA; 4 Structural Biology Science Technology Platform, The Francis Crick Institute, London, UK; 5 Institute of Structural and Molecular Biology, Birkbeck College London, London, UK; 6 Present Address: Department of Plant Biotechnology and Bioinformatics, Ghent University – Center for Plant Systems Biology, Flemish Institute for Biotechnology, Ghent, Belgium

**Keywords:** Transport carrier, Coat complexes, Cryoelectron tomography, Endoplasmic reticulum

Correction to: *Nature Cell Biology* https://doi.org/10.1038/s41556-026-01964-2, published online 20 May 2026.

The authors wish to correct the article to acknowledge a relevant recent article by Nair et al.^1^, which is now cited in the Discussion. The HTML and PDF versions of the article have now been corrected.
