# Supplementary material for: In situ cryo-ET defines the ultrastructure of ER exit sites in human cells
Source: Nat Cell Biol. 2026 May 20;28(6):1258–68. doi: 10.1038/s41556-026-01964-2 (PMC13279273; doi:10.1038/s41556-026-01964-2)
Supplement: Supplementary file 1 — Reporting Summary [file 41556_2026_1964_MOESM1_ESM.pdf]

nature portfolio

Corresponding author(s): Giulia Zanetti

Last updated by author(s): Apr 3, 2026

Reporting Summary

Nature Portfolio wishes to improve the reproducibility of the work that we publish. This form provides structure for consistency and transparency in reporting. For further information on Nature Portfolio policies, see our Editorial Policies and the Editorial Policy Checklist.

Statistics

For all statistical analyses, confirm that the following items are present in the figure legend, table legend, main text, or Methods section.

n/a	Confirmed	
☐	☒	The exact sample size (*n*) for each experimental group/condition, given as a discrete number and unit of measurement
☐	☒	A statement on whether measurements were taken from distinct samples or whether the same sample was measured repeatedly
☐	☒	The statistical test(s) used AND whether they are one- or two-sided<br>*Only common tests should be described solely by name; describe more complex techniques in the Methods section.*
☒	☐	A description of all covariates tested
☐	☒	A description of any assumptions or corrections, such as tests of normality and adjustment for multiple comparisons
☐	☒	A full description of the statistical parameters including central tendency (e.g. means) or other basic estimates (e.g. regression coefficient) AND variation (e.g. standard deviation) or associated estimates of uncertainty (e.g. confidence intervals)
☐	☒	For null hypothesis testing, the test statistic (e.g. *F*, *t*, *r*) with confidence intervals, effect sizes, degrees of freedom and *P* value noted<br>*Give P values as exact values whenever suitable.*
☒	☐	For Bayesian analysis, information on the choice of priors and Markov chain Monte Carlo settings
☒	☐	For hierarchical and complex designs, identification of the appropriate level for tests and full reporting of outcomes
☐	☒	Estimates of effect sizes (e.g. Cohen's *d*, Pearson's *r*), indicating how they were calculated

Our web collection on statistics for biologists contains articles on many of the points above.

Software and code

Policy information about availability of computer code

Data collection	No non-standard software was used to collect the data presented in this manuscript.
Data analysis	All novel code used in this manuscript can be found here: https://github.com/KTDownes/Multi-scale-Molecular-Imaging-of-Human-Cells-reveals-COPI-and-COPII-Vesicles-at-ER-Exit-Sites<br><br>Commercial software for data analysis includes GraphPad Prism and Imaris Microscopy Image Analysis Software (Oxford Instruments). All distance analyses were performed using the Shortest Distance calculation in Imaris, as detailed in the Materials and Methods. To create linescans, individuals linescans were generated in Imaris. The data from at least 30 structures was exported and used to calculate a representative linescan via degree 2 local polynomial regression, using the loess function from the stats package (v4.3.3) in R (v4.3.3).<br>To perform ribosome subtomogram averaging, we used WARP/M software (https://warpem.github.io/warp/) and relion4.0

For manuscripts utilizing custom algorithms or software that are central to the research but not yet described in published literature, software must be made available to editors and reviewers. We strongly encourage code deposition in a community repository (e.g. GitHub). See the Nature Portfolio guidelines for submitting code & software for further information.

Data

Policy information about availability of data

All manuscripts must include a data availability statement. This statement should provide the following information, where applicable:

- Accession codes, unique identifiers, or web links for publicly available datasets
- A description of any restrictions on data availability
- For clinical datasets or third party data, please ensure that the statement adheres to our policy

Raw data for all ERES tomograms are have been deposited in the EMPIAR database (EMPIAR 13270). The two representative tomograms shown in Fig. 3 have been deposited in the EMDB database (EMD-56606 and EMD-56951). The ribosome subtomogram averaging map has been deposited in the EMDB database (EMD-56963).
Source data have been provided in Source Data. All other data supporting the findings of this study are available from the corresponding author on reasonable request.

Research involving human participants, their data, or biological material

Policy information about studies with human participants or human data. See also policy information about sex, gender (identity/presentation), and sexual orientation and race, ethnicity and racism.

Reporting on sex and gender	This study uses the immortalised human cell line HTERT-1 RPE-1 which are female.
Reporting on race, ethnicity, or other socially relevant groupings	*Please specify the socially constructed or socially relevant categorization variable(s) used in your manuscript and explain why they were used. Please note that such variables should not be used as proxies for other socially constructed/relevant variables (for example, race or ethnicity should not be used as a proxy for socioeconomic status).* *Provide clear definitions of the relevant terms used, how they were provided (by the participants/respondents, the researchers, or third parties), and the method(s) used to classify people into the different categories (e.g. self-report, census or administrative data, social media data, etc.)* *Please provide details about how you controlled for confounding variables in your analyses.*
Population characteristics	*Describe the covariate-relevant population characteristics of the human research participants (e.g. age, genotypic information, past and current diagnosis and treatment categories). If you filled out the behavioural & social sciences study design questions and have nothing to add here, write "See above."*
Recruitment	*Describe how participants were recruited. Outline any potential self-selection bias or other biases that may be present and how these are likely to impact results.*
Ethics oversight	*Identify the organization(s) that approved the study protocol.*

Note that full information on the approval of the study protocol must also be provided in the manuscript.

Field-specific reporting

Please select the one below that is the best fit for your research. If you are not sure, read the appropriate sections before making your selection.

☒ Life sciences ☐ Behavioural & social sciences ☐ Ecological, evolutionary & environmental sciences

For a reference copy of the document with all sections, see nature.com/documents/nr-reporting-summary-flat.pdf

Life sciences study design

All studies must disclose on these points even when the disclosure is negative.

Sample size	We performed multiple rounds of in-situ cryo-FIBSEM and cryo-ET in our search for ER exit sites which ultimately yielded a total of 63 COPII-containing cryo-tomograms. From these, we extracted and analysed a total of 344 coated events (95 COPII vesicles, 62 COPII buds, 39 COPI vesicles, and 148 COPI buds). For ribosome subtomogram avearging, 16543 ribosome particles were included. For light microscopy analysis no statistical method was used to determine sample size. Instead, sample sizes were determined based on previous work.
Data exclusions	Tilt series with poor alignment quality were removed, however this is well established and accepted within the field. Due to the remit of this paper, only tomograms containing areas of interest were analysed using the processing pipeline outlined in the methods. For the morphology analysis when a coated structure was only partially captured by the field of view of the tomogram and therefore its identity as a vesicle or bud was ambiguous, said structure was excluded from the morphology analysis. For light microscopy analysis no data were intentionally excluded. Random structures and cells were chosen for analysis.
Replication	The tomogram data presented in this paper are derived from a total of 25 cells across 5 cryo-FIBSEM and cryo-ET experiments. For the light microscopy experiments replication details are provided in the figure legends and methods section. All experiments were conducted in duplicate or triplicate. STED experiments were repeated once. Each experiment consisted of 5-10 biological repeats.

Randomization	Ribosome particles falling into the two halves for independent processing were selected randomly. Cells were randomly chosen for light microscopy imaging without bias and punctate structures were chosen for linescan analysis without bias.
Blinding	Not relevant as there were no treatment groups.

Reporting for specific materials, systems and methods

We require information from authors about some types of materials, experimental systems and methods used in many studies. Here, indicate whether each material, system or method listed is relevant to your study. If you are not sure if a list item applies to your research, read the appropriate section before selecting a response.

Materials & experimental systems

n/a	Involved in the study	
☐	☒	Antibodies
☐	☒	Eukaryotic cell lines
☒	☐	Palaeontology and archaeology
☒	☐	Animals and other organisms
☒	☐	Clinical data
☒	☐	Dual use research of concern
☒	☐	Plants

Methods

n/a	Involved in the study	
☒	☐	ChIP-seq
☒	☐	Flow cytometry
☒	☐	MRI-based neuroimaging

Antibodies

Antibodies used	Sec16A (Bethyl Laboratories; A300-648A, RRID:AB_519338), Sec31A (BD Sciences; 612351, RRID:AB_399717), TFG (Novus Biologicals; NBP2-62212), ERGIC-53 (Santa Cruz Biotechnology; sc-66880, RRID:AB_2136001), and COPB (Santa Cruz Biotechnology; sc-393615)
Validation	All antibodies used are commercially available and complete validation information can be found on the respective suppliers website.<br>Sec16A - https://www.fortislife.com/products/primary-antibodies/rabbit-anti-kiaa0310-antibody/BETHYL-A300-648?selected=A300-648A<br>Sec31A - https://www.bdbiosciences.com/en-gb/products/reagents/western-blotting-and-molecular-reagents/western-blot-reagents/purified-mouse-anti-sec31a.612351?tab=product_details<br>TFG - https://www.novusbio.com/products/tfg-antibody-tfg-03_nbp2-62212<br>ERGIC-53 - https://www.scbt.com/p/ergic-53-antibody-h-245?srsltid=AfmBOopo29_9vXzYFSMLGVis2im19wg8SMU044bFDdywHJOugq36ANcx<br>COPB - https://www.scbt.com/p/copb-antibody-d-10

Eukaryotic cell lines

Policy information about cell lines and Sex and Gender in Research

Cell line source(s)	Human retinal pigmented epithelial cells (RPE-1) were obtained from ATCC.<br>CRISPR modified Halo-Sec23A and SNAP-tag-ERGIC-53 RPE-1 cells were sourced as described in https://doi.org/10.1016/j.celrep.2023.112635.. All lines were derived from the immortalised human HTERT-RPE-1 cell line which is female.
Authentication	This study uses human hTERT-immortalized RPE1 cells (CRL-4000 from ATCC; sex: female), which have been authenticated using STR profiling analysis. HaloTag-Sec23 and SnapTag-ERGIC53 were previously published in https://doi.org/10.1016/j.celrep.2023.112635. The validation included genomic DNA sequencing and western blot analysis.
Mycoplasma contamination	All cell lines tested negative for mycoplasma.
Commonly misidentified lines<br>(See ICLAC register)	none

Plants

Seed stocks	*Report on the source of all seed stocks or other plant material used. If applicable, state the seed stock centre and catalogue number. If plant specimens were collected from the field, describe the collection location, date and sampling procedures.*
Novel plant genotypes	*Describe the methods by which all novel plant genotypes were produced. This includes those generated by transgenic approaches, gene editing, chemical/radiation-based mutagenesis and hybridization. For transgenic lines, describe the transformation method, the number of independent lines analyzed and the generation upon which experiments were performed. For gene-edited lines, describe the editor used, the endogenous sequence targeted for editing, the targeting guide RNA sequence (if applicable) and how the editor was applied.*
Authentication	*Describe any authentication procedures for each seed stock used or novel genotype generated. Describe any experiments used to assess the effect of a mutation and, where applicable, how potential secondary effects (e.g. second site T-DNA insertions, mosiacism, off-target gene editing) were examined.*
