# Supplementary material for: In situ cryo-ET defines the ultrastructure of ER exit sites in human cells
Source: Nat Cell Biol. 2026 May 20;28(6):1258–68. doi: 10.1038/s41556-026-01964-2 (PMC13279273; doi:10.1038/s41556-026-01964-2)
Supplement: Supplementary file 2 — Peer Review file [file 41556_2026_1964_MOESM2_ESM.pdf]

Peer Review File

In situ cryo-ET defined the ultrastructure of ER exit sites in human cells

Corresponding Author: Dr Giulia Zanetti

Version 0:

Decision Letter:

*Please delete the link to your author homepage if you wish to forward this email to co-authors.

Dear Dr Zanetti,

Your manuscript, "Multi-scale Molecular Imaging of Human Cells reveals COPI and COPII Vesicles at ER Exit Sites", has now been seen by 3 referees, who are experts in cryo FIB-SEM and cryoEM (referee 1); cryoET, cryoCLEM and membrane contact sites (referee 2); and imaging, ER and secretion (referee 3). As you will see from their comments (attached below) they find this work of potential interest, but have raised substantial concerns, which in our view would need to be addressed with considerable revisions before we can consider publication in Nature Cell Biology.

Nature Cell Biology editors discuss the referee reports in detail within the editorial team, including the chief editor, to identify key referee points that should be addressed with priority, and requests that are overruled as being beyond the scope of the current study. To guide the scope of the revisions, I have listed these points below. We are committed to providing a fair and constructive peer-review process, so please feel free to contact me if you would like to discuss any of the referee comments further.

In particular, please focus your revision on providing

+ dynamic assessment of vesicle budding

+ clarity on potential artifacts, limitations and biases

+ quantification and controls

+ All other referee concerns pertaining to strengthening existing data, methodological details, clarifications and textual changes, should also be addressed.

+ Finally please pay close attention to our guidelines on statistical and methodological reporting (listed below) as failure to do so may delay the reconsideration of the revised manuscript. In particular please provide:

- a Supplementary Figure including unprocessed images of all gels/blots in the form of a multi-page pdf file. Please ensure that blots/gels are labeled and the sections presented in the figures are clearly indicated.

- a Supplementary Table including all numerical source data in Excel format, with data for different figures provided as different sheets within a single Excel file. The file should include source data giving rise to graphical representations and statistical descriptions in the paper and for all instances where the figures present representative experiments of multiple independent repeats, the source data of all repeats should be provided.

We would be happy to consider a revised manuscript that would satisfactorily address these points, unless a similar paper is published elsewhere, or is accepted for publication in Nature Cell Biology in the meantime.

When revising the manuscript please:

- ensure that it conforms to our format instructions and publication policies (see below and https://www.nature.com/nature/for-authors).

- provide a point-by-point rebuttal to the full referee reports verbatim, as provided at the end of this letter.

- for any revision that includes light microscopy data, we ask our authors to please include a completed light microscopy reporting table https://www.nature.com/documents/Light_microscopy_reporting_table.xlsx to ensure the methods are described thoroughly. The table will be available to reviewers and ultimately published should the manuscript be accepted at the journal.

- provide the completed Reporting Summary (found here https://www.nature.com/documents/nr-reporting-summary.pdf). This is essential for reconsideration of the manuscript will be available to editors and referees in the event of peer review. For more information see http://www.nature.com/authors/policies/availability.html or contact me.

When submitting the revised version of your manuscript, please pay close attention to our href="https://www.nature.com/nature-portfolio/editorial-policies/image-integrity">Digital Image Integrity Guidelines. and to the following points below:

-- that unprocessed scans are clearly labelled and match the gels and western blots presented in figures.
-- that control panels for gels and western blots are appropriately described as loading on sample processing controls
-- all images in the paper are checked for duplication of panels and for splicing of gel lanes.

Finally, please ensure that you retain unprocessed data and metadata files after publication, ideally archiving data in perpetuity, as these may be requested during the peer review and production process or after publication if any issues arise.

Nature Cell Biology is committed to improving transparency in authorship. As part of our efforts in this direction, we are now requesting that all authors identified as ‘corresponding author’ on published papers create and link their Open Researcher and Contributor Identifier (ORCID) with their account on the Manuscript Tracking System (MTS), prior to acceptance. ORCID helps the scientific community achieve unambiguous attribution of all scholarly contributions. You can create and link your ORCID from the home page of the MTS by clicking on ‘Modify my Springer Nature account’. For more information please visit please visit www.springernature.com/orcid.

This journal strongly supports public availability of data. Please place the data used in your paper into a public data repository, or alternatively, present the data as Supplementary Information. If data can only be shared on request, please explain why in your Data Availability Statement, and also in the correspondence with your editor. Please note that for some data types, deposition in a public repository is mandatory - more information on our data deposition policies and available repositories appears below.

Please submit the revised manuscript files and the point-by-point rebuttal to the referee comments using this link:

Link Redacted

*This url links to your confidential home page and associated information about manuscripts you may have submitted or be reviewing for us. If you wish to forward this email to co-authors, please delete the link to your homepage.

We would like to receive a revised submission within six months.

We hope that you will find our referees' comments, and editorial guidance helpful. Please do not hesitate to contact me if there is anything you would like to discuss.

Best wishes,

Angela Parrish

Angela R Parrish, PhD
Locum Senior Editor
Nature Cell Biology

Reviewers' Comments:

Reviewer #1 (Remarks to the Author):

Summary
Downes et al. present an overview of COPI and COPII trafficking using cryo-electron tomography to unpick the arrangement, organisation and interaction of vesicles between ER exit sites (ERES) and the ER golgi intermediate compartment (ERGIC), as well as the wider context of Golgi. The authors present beautiful tomographic datasets and segmentations that allow the network of COPII and COPI vesicles to be seen in and amongst adjacent membrane compartments. The use of Halo Tags

and Ligands in the context of live cell cryo electron tomography allows a wide range of questions to be addressed and presented here represents a simple elegant model to target ERES and ERGIC dynamics.
The authors use a low magnification (EM) step to help with fluorescence targeting which is technically novel and enriches the high magnification interpretation by widening the cellular observation field. While the identification and determination of size and shape the population of COPI and COPII vesicles is well established, the presentation of the data at time makes it hard to interpret how these sit into the wider context of the cellular tomograms.
Attempts are made to show this (Figure 3), but the difficulty in interpreting the figures makes the results hard to follow. While there is enough data to be able to understand that what they conclude is, based on probability, correct, an edit of the figures to better annotate and illustrate the complex 3D structures that are present within the data and their relevance to the overall ERES-ERGIC COPI/COPII trafficking would be very beneficial.
The manuscript at time fails to present quantitative data to back up statements, of which I have made comment below. Furthermore, in some sections of the result the descriptions of the EM are too technical for a journal such as Nature Cell Biology and are in some cases irrelevant and should form part of the methods section.
Overall, the manuscript presents a wonderful leap in potential in understanding ER-Golgi interaction dynamics on the molecular scale, and with some edits and further quantification would be a good addition to the Nature Cell Biology portfolio.

Comments:
Line 102 and 105 – “unambiguously”.
Lines 109 – 130 can be reduced in size, with some moved to the methods. I would condense this to focus on need to utilise a fluorescent model to increase the likelihood of encountering ERES targets and that they assessed the RPE-1 cell lines for its functionality and ability to (Figure 1 and S1). It should also be made clear that the cryoET approach requires one that is live-cell compatible.
Lines 131-149. This paragraph focuses too much on the technical details of the EM and correlation. The choice of pixel size referenced is based on the ambition to perform structural studies on the COP vesicles but is not realised. Therefore, this context is largely superfluous. The authors aim to build the difficulties in targeting accurately with fluorescence into their adoption of an intermediate magnification tilt series, which is of technical note however for the purposes of the scope of the paper, which is very much cell biology focused, the merits of the technical parts here are less relevant. I would greatly reduce this section to focus solely on how ERES sites were achieved from the context of the cryoET structural cell biology and mentioned a short phrase to state that fluorescence combined with a low mag tilt series was the key aspect that allowed targeting of sufficient examples for further analysis.
Line 141 – “restricted tilt series” what does “restricted” mean here?
Line 145 – 168. I do not see any reason for this to be included in the results here as it is mostly a methodological note with minimal relevance to the rest of what is written.
The portion on ribosomal structure determination should be written to reflect the ambition to work out ribosome-excluded regions of the data and quantitative measures of ERES localisation.
Line 201 – “Quantitative”. I find that in this section there is very little quantitation. IT would have been useful to quantitate the ribosome exclusion zone, especially with the extensive segmentation and particle position determination. Furthermore, how was the boundary delineated? A more thorough description/analysis would have provided insight into the global segregation of ERESs from the rest of the cytosolic constituents in a way that is less accessible to other techniques such as fluorescence.
Line 206 – “rather a sharp cut off” what does this phrase mean?
Figure 3. This figure is very hard to interpret and is referenced in many places as an example of, often, quite intricate discussion on morphology and COPI, COPII and other phenomena. There is no annotation of the figure to illustrate points made in the manuscript and the caption is not helpful. Relevant places where figure 3 is referenced are ribosome density (line 206), vesicular-tubular clusters (lines 227-8), non-coated structures (line 247), buds (line 257), and vesicle morphology (line 381). Displaying whole tomogram segmentations of 9 tomograms, even with 3 colours to delineate COPII, COP1 and ribosomes, is a very difficult way to represent such a diverse range of important structure relevant to the core of this paper. I implore the authors to rethink this figure to tease out (annotate, use of insets, panels etc) details as well as showing the global organisation. I simply got lost trying to investigate thing reference in the text.
Line 211-212. What is high resolution confocal fluorescence microscopy?
Line 217-219. Is this sentence not essentially saying the same thing twice? To what end?
Line 219 – “Of the latter” Delete.
Line 220 – Use of high resolution (again) should be removed.
Line 221 – How does the distance measurement of COPI to Sec23A+ puncta compare to the measurements in tomograms?
Line 222 – 77% of COPI puncta are not associated with COPII, but where is the quantification of the data presented? I could not find it, though I have to assume you had to count totals to calculate percentage.
Line 227-228 – It is hard to discern the observations made within figure 3.
Line 229. “High resolution” is used again.
Line 229-230 – Some quantification of relative positions observed would help here. Later in the paragraph, the fluorescence data (also in lines 231-233) are used to justify what the origin of COPI vesicles is within the tomography data (lines 233-235). It would as a reader to understand what prevalence there is in the data e.g. are COPI [, COPII] and ERGIC-53 always co-localised, majority of time, or less?
Line 236-237 – For COPII there are only 2 examples with more than 7 vesicles so that range 1-19 seems a little misleading (one could think it ranges all the way from 1 to 19, even though examples with 8 to 17 are not in the data). So it may be worth caveating the oddities in the range.
Line 250. Use of black star. I think it would be much more useful to excise this example as an inset and then 3D render the decoration with COPII across the vesicle surface to illustrate its “uncoating”. As is, it is small and unannotated. Alternatively, approaches such as surforama may allow a world map of distribution to be rendered (10.1101/2024.01.05.574336)
Line 252. What do the authors mean by “anisotropy of the reconstruction”?

Line 257-258 – Where are the data to back up this statement?
Line 268 - I could not see any quatification of the root mean square error related to the fit in Figure 5A.
Line 269. For the volume calculations, how did you account for the missing wedge?
Line 270. You give diameters based off the median volume, but how does the diameter compare to measurements from vesicles within the data? This would be good validation.
Line 278-279. As examples from tomography data are limited to Chlamydomonas and the mouse work is reconstituted (and therefore act as a contributory factor), use of the word species may be a bit strong and should be replaced to something along the line of phyla or kingdom (animals vs plants).
Line 281-282. As there are ~ 4 points responsible for the skew, it would be useful to have the STDEV and ERRORs in the plot.
How much does the 'restriction' of the low mag tilt series affect the interpretation of the organisation of the ERGIC, ERES and Golgi? If the tilt series is only at low angles, does this produce difficulties in segmenting and analysing in 3D?
Line 339. "Extending to 1.5nm resolution" Is this based from the ribosome reconstruction rather than the CTF fit?
Why was no comparison done for the TFG localisation with COPI?
Line 346-348 – Where are the data shown for resolving such tubules etc?
Line 371-373 – As cytoskeleton is discussed, it may be worth having representative images of what is discussed from the data (that filaments are not interacting with the ER-Golgi network).

Figures:
Figure 1. The use of a star in 1D should be referenced in the caption. As mentioned above, to help with understanding uncoating it would be helpful to tease this out in a separate figure or panel. If in this figure, a row or vesicle types as panel E at the bottom may be one way to show this.

Figure 2. There are 28 examples each of COPI and COPII. While it is interesting to see the diversity, I think it may be more worthwhile focusing on a subset here and then annotating the specifics in the difference in lattice organisation of COPI and COPII. The diversity may then potentially be shown as a supplement.

Figure 3. This needs to be properly reworked and annotated. The caption does not help. This figure is referenced four times in the text to illustrate several phenomena, and this could be presented appropriately to help the reader track what the different places in the text mean.

Supplementary Figure 4
As a further row, an enlarged, to-scale shot of the vesicle should be shown to contextualise the maps D-F.

Supplementary Figure 5
The ellipsoidal fit is not clear in A, and there is no quantification shown to back up the statement on line 268.

Reviewer #2 (Remarks to the Author):

This manuscript presents a thorough high-resolution description of the ER-ERGIC interface in human cells, with a focus on the arrangement of COPI and COPII at ER-exit sites. The authors use cryo-electron tomography combined with fluorescence microscopy. The results show very convincingly the occurrence of COPII coated vesicular carriers, a topic of recent debate. Particularly for this reason, but also for the large amount of other architectural details that lead to conclusions about dynamics, structure and mechanism, this study is of high interest for the cell biology and the membrane trafficking community. I therefore recommend publication of this manuscript, with some suggestions for revisions as follows.

Major:
1. Line 203 and following: It would be great to give some quantification of the percentage or number of vesicles vs. membrane buds already here, as it remains unclear how relevant the ultrastructure description is without numbers.

2. Line 216: Why was is not possible to quantify the displacement between ER and COPII vesicles from the cryo-ET data, to have a direct comparison with the confocal imaging data?

3. Line 194-195: The statement "This suggests that the coat architecture described previously at high resolution is likely to be physiologically relevant" seems an overstatement. I agree with everything else concluded in this paragraph, but the here presented data does not allow to conclude that the high-resolution structures were correct (even if they likely were).

4. Line 257-8: How can the authors be so certain in all cases about the assignment of ER, ERGIC and Golgi membranes? In particular as in lines 242-3 they say it's difficult to distinguish ERGIC and Golgi.

5. Line 267: Could the apparent ellipsoidity of the vesicles be influenced by the anisotropic resolution / the missing wedge of the tomograms? How can this be excluded?

6. Lines 294-297: How different is this analysis from the one of ellipsoid shape mentioned above, and why are they referred

to in different parts of the manuscript?

7. The success rate of the CLEM protocol, especially the initial variant with directly acquiring high-magnification tomograms, seems very poor. Can the authors exclude that this is not only due to difficulties in alignments, but perhaps also due to false-positive signals, including accumulations of labelled Sec23 that is not assembled on membranes?

8. Along the same lines, in line 152/153 the authors that even with the optimised CLEM pipeline, 72% of regions of interest contained non-coated membranes. Is it possible to give an estimate of how certainly these did not contain Halo-labelled Sec23? And for the COPI containing tomograms mentioned in lines 223/224, how certain is it that these did not contain any Halo-labelled Sec23?

9. Since the template matching for COPI and COPII worked well, why was the data not amenable to subtomogram averaging, as mentioned in the Discussion lines 384-385?

Minor points:
10. Abstract: line 22: It is clear what "unperturbed" means. In fact, the cells are CRISPR edited, does that qualify as unperturbed? Or do the authors mean no drug treatment?

11. Line 42-44: It would be good to back up this sentence with a reference.

12. Line 84-85: I don't seem to understand the sentence starting with "Live cell imaging…" e.g. what do you mean by steady state location?

13. In Figure 2, are the images showing in vitro vesicles shown at exactly the same scale as the others? If yes, please indicate in legend, if not, please make extra scale bars.

14. Please add numbers n to the legend of Figure 5 panels. In particular, panel D, E and F would benefit from indicating what n membranes or vesicles were analysed, and what n tomograms they came from (I know this info is in the text, but it's difficult to access when looking at the figure)

15. In the Results section, past and present tense are used inconsistently. For example, at about line 166 there is a sharp change to present tense.

16. In Supplementary Figure 8, I believe the label next to the purple vesicle should be COPI (not COPII).

Reviewer #3 (Remarks to the Author):

Downes et al. present an exciting set of experiments using cryo-correlative fluorescence to address arguably the most contentious question today in secretory pathway biology: the nature of the ER exit site (ERES). They use a newly emerging tool in cryo-(high resolution template matching, HRTM) to find the structures of COPI and COPII in lamella cut through putative ER exit sites, as identified using pre- and post-lamella cutting cryo-fluorescence from an endogenously-tagged RPE1 line. They further support this with some quantitative localization studies using STED microscopy to try to address the underlying reasons for some of what is observed in the cryoET data. This paper represents extremely laborious and expensive approaches, and I appreciate the significant effort the authors have applied to try solve one of the biggest questions in cell biology today.
In principle, this ought to be the landmark paper that finally puts the issue of ER exit site (ERES) structure to rest, as the only way I can even imagine this question being directly addressed is the approach presented here. However, the data as it is presented sort of drops the ball on this. I don't have any skin in this game, and even I am not clearly convinced by what is shown in the figures, despite some very aggressive phrasing on the part of the authors: "our results clearly and unambiguously support the vesicular transport model." If the authors really have the data to back this up, I will change my opinion, but as it is presented now this will be another in a long list of papers making big claims about mapping the mechanisms of ER exit without actually backing it up with their data. I have detailed my major and minor issues below.
Major concerns:
1. The authors claim several times in the text that they can unequivocally identify vesicles as opposed tubular structures. I am confused why they have never shown them? Every "vesicle" I can see easily in the figures is truncated on one side or the other, and I do not see how they could possibly know unequivocally that this is a vesicle rather than a tube, especially given that the both theoretical and the most recent works arguing for tubes already stated that they frequently see pearling structures, which would appear as a vesicle if truncated in this way (see PMIDs: 33852913, 14645071, for example).
2. In general, I applaud the use of the emerging technique of template matching, but it comes with a risk that must be carefully modulated. Namely, it biases towards seeing what is used as the bait. Since the authors have used in vitro-derived COPII structures on vesicles as their bait, this runs the risk of only finding COPII in regions where it approximates a spherical structure. This would explain why they see such an imbalance between COPI and COPII on budding vs vesicular structures, since the structure of the inner coat is more well-defined. The use of pattern matching on "un-coated structures" is not an acceptable control for this, the authors need to evaluate how "flat" a structure can be before they fail to resolve it and think of

an appropriate control for a) where they could be missing COPII and b) how often it can erroneously match a coated bud (seems unlikely, but this should be checked). At the very least, this needs a more significant treatment on the method that can allow a reader to critically evaluate this potential limitation. I am concerned this has biased the result to give exactly what the authors expect to see, rather than a fair evaluation of the data.
3. I'm sorry to the authors because I know this is a hard critique to evaluate, but given the importance of this work and the boldness of the claims, the authors need to directly address the potential of vesiculation artifacts as a result of freezing. Plunge freezing is a challenging pipeline to execute for fragile membrane structures like this, if the cells have contacted the air for too long during blotting, it is well established in the field that thin tubules will vesiculate from transient osmotic stress. Obviously, this has never been applied to exit sites, where we are not sure of the structure, but numerous people in the field have described this for thin peripheral ER tubules and sorting tubules on endosomes. The best way to verify that the freezing process was non-perturbative is to find an example of an endosome within one of the lamella and show that the tubules are intact and not fragmented, since the peripheral ER tubules will be hard to get in lamella and may respond differently to osmotic stress that things in the perinuclear region. Without some control of this type, I doubt the advocates for the lateral diffusion of proteins into the exit site will accept the results of the paper, and the controversy will continue.
4. I am concerned by the lack of the authors to find microtubules in the vicinity of ERES, since those were so prevalent in the whole cell FIB-SEM volumes published. This begs one of two possibilities: A) The lamella selection and validation is biasing the presented results towards ERES that do not have microtubules present. This is potentially a problem for the interpretations of the paper. B) The nature of the lamella cutting is such that it nearly always removes the microtubule in proximity due to some inherent angling of the two with respect to one another and the angle of the FIB milling beam. This would be interesting, and is not a problem for the paper's interpretations, per se. This should be addressed, if possible.
5. Throughout the paper, the authors interpret "budding" vesicles as though they have captured a stage in a dynamic process. The data presented is static, of course, I do not think the authors can make this kind of inference from it. I recognize that interpreting the data this way amplifies the perceived impact, but if the authors cannot find a way to support their interpretations with something about the dynamic processes at this spot, I think they cannot make such firm statements. Is it possible their existing STED or NSPARC experimental pipeline could help with this? Live imaging of the recovery from bleaching, etc. might help to address tis directly.
Minor Points:
1. The methods section is insufficiently detailed for me to critically evaluate some of the technical aspects of the paper. Some effort contributed to supplementary figures to illustrate the mechanics would go a long way in establishing trust in the approach. It would make it much easier if the code used to perfume the template matching, segmentation, and STED image analysis was provided.
2. Are channel alignments performed for the STED imaging using fiducial markers? I can't find this explicitly written anywhere.
3. Can the authors provide maps of where the lamella were cut in each cell in the supplement? For readers who frequently do EM, this would help to understand what membranes are visible in the tomograms. This could also potentially affect why the authors' results are potentially at odds with some published work, where the techniques used only work in the furthest edges of cells (e.g. 33852719). It's formally possible that both mechanisms are valid, but differentially applied based on subcellular location or polarity.

GUIDELINES FOR MANUSCRIPT SUBMISSION TO NATURE CELL BIOLOGY

READABILITY OF MANUSCRIPTS – Nature Cell Biology is read by cell biologists from diverse backgrounds, many of whom are not native English speakers. Authors should aim to communicate their findings clearly, explaining technical jargon that might be unfamiliar to non-specialists, and avoiding non-standard abbreviations. Titles and abstracts should concisely communicate the main findings of the study, and the background, rationale, results and conclusions should be clearly explained in the manuscript in a manner accessible to a broad cell biology audience. Nature Cell Biology uses British spelling.

MANUSCRIPT FORMAT – please follow the guidelines listed in our Guide to Authors regarding manuscript formats at Nature Cell Biology.

TITLE – should be no more than 100 characters including spaces, without punctuation and avoiding technical terms, abbreviations, and active verbs..

AUTHOR NAMES – should be given in full.

AUTHOR AFFILIATIONS – should be denoted with numerical superscripts (not symbols) preceding the names. Full addresses should be included, with US states in full and providing zip/post codes. The corresponding author is denoted by: "Correspondence should be addressed to [initials]."

ABSTRACT AND MAIN TEXT – please follow the guidelines that are specific to the format of your manuscript, as listed in

our Guide to Authors (http://www.nature.com/ncb/pdf/ncb_gta.pdf) Briefly, Nature Cell Biology Articles, Resources and Technical Reports have 3500 words, including a 150 word abstract, and the main text is subdivided in Introduction, Results, and Discussion sections. Nature Cell Biology Letters have up to 2500 words, including a 180 word introductory paragraph (abstract), and the text is not subdivided in sections.

ACKNOWLEDGEMENTS – should be kept brief. Professional titles and affiliations are unnecessary. Grant numbers can be listed.

AUTHOR CONTRIBUTIONS – must be included after the Acknowledgements, detailing the contributions of each author to the paper (e.g. experimental work, project planning, data analysis etc.). Each author should be listed by his/her initials.

FINANCIAL AND NON-FINANCIAL COMPETING INTERESTS – the authors must include one of three declarations: (1) that they have no financial and non-financial competing interests; (2) that they have financial and non-financial competing interests; or (3) that they decline to respond, after the Author Contributions section. This statement will be published with the article, and in cases where financial and non-financial competing interests are declared, these will be itemized in a web supplement to the article. For further details please see https://www.nature.com/licenceforms/nrg/competing-interests.pdf.

REFERENCES – are limited to a total of 70 for Articles, Resources, Technical Reports; and 40 for Letters. This includes references in the main text and Methods combined. References must be numbered sequentially as they appear in the main text, tables and figure legends and Methods and must follow the precise style of Nature Cell Biology references. References only cited in the Methods should be numbered consecutively following the last reference cited in the main text. References only associated with Supplementary Information (e.g. in supplementary legends) do not count toward the total reference limit and do not need to be cited in numerical continuity with references in the main text. Only published papers can be cited, and each publication cited should be included in the numbered reference list, which should include the manuscript titles. Footnotes are not permitted.

METHODS – Nature Cell Biology publishes methods online. The methods section should be provided as a separate Word document, which will be copyedited and appended to the manuscript PDF, and incorporated within the HTML format of the paper.

Methods should be written concisely, but should contain all elements necessary to allow interpretation and replication of the results. As a guideline, Methods sections typically do not exceed 3,000 words. The Methods should be divided into subsections listing reagents and techniques. When citing previous methods, accurate references should be provided and any alterations should be noted. Information must be provided about: antibody dilutions, company names, catalogue numbers and clone numbers for monoclonal antibodies; sequences of RNAi and cDNA probes/primers or company names and catalogue numbers if reagents are commercial; cell line names, sources and information on cell line identity and authentication. Animal studies and experiments involving human subjects must be reported in detail, identifying the committees approving the protocols. For studies involving human subjects/samples, a statement must be included confirming that informed consent was obtained. Statistical analyses and information on the reproducibility of experimental results should be provided in a section titled “Statistics and Reproducibility”.

All Nature Cell Biology manuscripts submitted on or after March 21 2016 must include a Data availability statement as a separate section after Methods but before references, under the heading "Data Availability”. . For Springer Nature policies on data availability see http://www.nature.com/authors/policies/availability.html; for more information on this particular policy see http://www.nature.com/authors/policies/data/data-availability-statements-data-citations.pdf. The Data availability statement should include:

• Accession codes for primary datasets (generated during the study under consideration and designated as "primary accessions") and secondary datasets (published datasets reanalysed during the study under consideration, designated as "referenced accessions"). For primary accessions data should be made public to coincide with publication of the manuscript. A list of data types for which submission to community-endorsed public repositories is mandated (including sequence, structure, microarray, deep sequencing data) can be found here http://www.nature.com/authors/policies/availability.html#data.

• Unique identifiers (accession codes, DOIs or other unique persistent identifier) and hyperlinks for datasets deposited in an approved repository, but for which data deposition is not mandated (see here for details http://www.nature.com/sdata/data-policies/repositories).

• At a minimum, please include a statement confirming that all relevant data are available from the authors, and/or are included with the manuscript (e.g. as source data or supplementary information), listing which data are included (e.g. by figure panels and data types) and mentioning any restrictions on availability.

• If a dataset has a Digital Object Identifier (DOI) as its unique identifier, we strongly encourage including this in the Reference list and citing the dataset in the Methods.

We recommend that you upload the step-by-step protocols used in this manuscript to protocols.io. More details can found at https://www.protocols.io/help/publish-articles.

DISPLAY ITEMS – main display items are limited to 6-8 main figures and/or main tables for Articles, Resources, Technical Reports; and 5 main figures and/or main tables for Letters. For Supplementary Information see below.

FIGURES – Colour figure publication costs $600 for the first, and $300 for each subsequent colour figure. All panels of a multi-panel figure must be logically connected and arranged as they would appear in the final version. Unnecessary figures and figure panels should be avoided (e.g. data presented in small tables could be stated briefly in the text instead).

All imaging data should be accompanied by scale bars, which should be defined in the legend.
Cropped images of gels/blots are acceptable, but need to be accompanied by size markers, and to retain visible background signal within the linear range (i.e. should not be saturated). The boundaries of panels with low background have to be demarked with black lines. Splicing of panels should only be considered if unavoidable, and must be clearly marked on the figure, and noted in the legend with a statement on whether the samples were obtained and processed simultaneously. Quantitative comparisons between samples on different gels/blots are discouraged; if this is unavoidable, it should only be performed for samples derived from the same experiment with gels/blots were processed in parallel, which needs to be stated in the legend.

Figures should be provided at approximately the size that they are to be printed at (single column is 86 mm, double column is 170 mm) and should not exceed an A4 page (8.5 x 11"). Reduction to the scale that will be used on the page is not necessary, but multi-panel figures should be sized so that the whole figure can be reduced by the same amount at the smallest size at which essential details in each panel are visible. In the interest of our colour-blind readers we ask that you avoid using red and green for contrast in figures. Replacing red with magenta and green with turquoise are two possible colour-safe alternatives. Lines with widths of less than 1 point should be avoided. Sans serif typefaces, such as Helvetica (preferred) or Arial should be used. All text that forms part of a figure should be rewritable and removable.

We accept files from the following graphics packages in either PC or Macintosh format:

- For line art, graphs, charts and schematics we prefer Adobe Illustrator (.AI), Encapsulated PostScript (.EPS) or Portable Document Format (.PDF). Files should be saved or exported as such directly from the application in which they were made, to allow us to restyle them according to our journal house style.

- We accept PowerPoint (.PPT) files if they are fully editable. However, please refrain from adding PowerPoint graphical effects to objects, as this results in them outputting poor quality raster art. Text used for PowerPoint figures should be Helvetica (preferred) or Arial.

- We do not recommend using Adobe Photoshop for designing figures, but we can accept Photoshop generated (.PSD or .TIFF) files only if each element included in the figure (text, labels, pictures, graphs, arrows and scale bars) are on separate layers. All text should be editable in ‘type layers’ and line-art such as graphs and other simple schematics should be preserved and embedded within 'vector smart objects’ - not flattened raster/bitmap graphics.

- Some programs can generate Postscript by 'printing to file' (found in the Print dialogue). If using an application not listed above, save the file in PostScript format or email our Art Editor, Allen Beattie for advice (a.beattie@nature.com).

Regardless of format, all figures must be vector graphic compatible files, not supplied in a flattened raster/bitmap graphics format, but should be fully editable, allowing us to highlight/copy/paste all text and move individual parts of the figures (i.e. arrows, lines, x and y axes, graphs, tick marks, scale bars etc.). The only parts of the figure that should be in pixel raster/bitmap format are photographic images or 3D rendered graphics/complex technical illustrations.

All placed images (i.e. a photo incorporated into a figure) should be on a separate layer and independent from any superimposed scale bars or text. Individual photographic images must be a minimum of 300+ DPI (at actual size) or kept constant from the original picture acquisition and not decreased in resolution post image acquisition. All colour artwork should be RGB format.

EXTENDED DATA FIGURES - When re-submitting your manuscript, please ensure that any supplementary figures and tables that are crucial to the manuscript’s conclusions are converted into Extended Data figures and tables to increase visibility of these data. Extended Data figures and tables are online-only (present in the online PDF and full-text HTML versions of the paper), peer-reviewed display items that provide essential background to the article but are not included in the main article due to space constraints. A maximum of ten Extended Data display items (figures and tables) is permitted.

FIGURE LEGENDS – must not exceed 350 words for each figure to allow fit on a single printed NCB page together with the figure. They must include a brief title for the whole figure, and short descriptions of each panel with definitions of the symbols used, but without detailing methodology.

TABLES – main tables should be provided as individual Word files, together with a brief title and legend. For supplementary tables see below.

SUPPLEMENTARY INFORMATION – Supplementary information is material directly relevant to the conclusion of a paper, but which cannot be included in the printed version in order to keep the manuscript concise and accessible to the general reader. Supplementary information is an integral part of a Nature Cell Biology publication and should be prepared and presented with as much care as the main display item, but it must not include non-essential data or text, which may be

removed at the editor's discretion. All supplementary material is fully peer-reviewed and published online as part of the HTML version of the manuscript. Supplementary Figures and Supplementary Notes are appended at the end of the main PDF of the published manuscript.

Supplementary items should relate to a main text figure, wherever possible, and should be mentioned sequentially in the main manuscript, designated as Supplementary Figure, Table, Video, or Note, and numbered continuously (e.g. Supplementary Figure 1, Supplementary Figure 2, Supplementary Table 1, Supplementary Table 2 etc.).

Unprocessed scans of all key data generated through electrophoretic separation techniques need to be presented in a supplementary figure that should be labelled and numbered as the final supplementary figure, and should be mentioned in every relevant figure legend. This figure does not count towards the total number of figures and is the only figure that can be displayed over multiple pages, but should be provided as a single file, in PDF or TIFF format. Data in this figure can be displayed in a relatively informal style, but size markers and the figures panels corresponding to the presented data must be indicated.

The total number of Supplementary Figures (not including the “unprocessed scans” Supplementary Figure) should not exceed the number of main display items (figures and/or tables (see our Guide to Authors and March 2012 editorial http://www.nature.com/ncb/authors/submit/index.html#suppinfo; http://www.nature.com/ncb/journal/v14/n3/index.html#ed). No restrictions apply to Supplementary Tables or Videos, but we advise authors to be selective in including supplemental data.

Each Supplementary Figure should be provided as a single page and as an individual file in one of our accepted figure formats and should be presented according to our figure guidelines (see above). Supplementary Tables should be provided as individual Excel files. Supplementary Videos should be provided as .avi or .mov files up to 50 MB in size. Supplementary Figures, Tables and Videos much be accompanied by a separate Word document including titles and legends.

GUIDELINES FOR EXPERIMENTAL AND STATISTICAL REPORTING

REPORTING REQUIREMENTS – We are trying to improve the quality of methods and statistics reporting in our papers. To that end, we are now asking authors to complete a reporting summary that collects information on experimental design and reagents. The Reporting Summary can be found here https://www.nature.com/documents/nr-reporting-summary.pdf)If you would like to reference the guidance text as you complete the template, please access these flattened versions at http://www.nature.com/authors/policies/availability.html.

STATISTICS – Wherever statistics have been derived the legend needs to provide the n number (i.e. the sample size used to derive statistics) as a precise value (not a range), and define what this value represents. Error bars need to be defined in the legends (e.g. SD, SEM) together with a measure of centre (e.g. mean, median). Box plots need to be defined in terms of minima, maxima, centre, and percentiles. Ranges are more appropriate than standard errors for small data sets. Wherever statistical significance has been derived, precise p values need to be provided and the statistical test used needs to be stated in the legend. Statistics such as error bars must not be derived from n<3. For sample sizes of n<5 please plot the individual data points rather than providing bar graphs. Deriving statistics from technical replicate samples, rather than biological replicates is strongly discouraged. Wherever statistical significance has been derived, precise p values need to be provided and the statistical test stated in the legend.

Information on how many times each experiment was repeated independently with similar results needs to be provided in the legends and/or Methods for all experiments, and in particular wherever representative experiments are shown.

We strongly recommend the presentation of source data for graphical and statistical analyses as a separate Supplementary Table, and request that source data for all independent repeats are provided when representative experiments of multiple independent repeats, or averages of two independent experiments are presented. This supplementary table should be in Excel format, with data for different figures provided as different sheets within a single Excel file. It should be labelled and numbered as one of the supplementary tables, titled “Statistics Source Data”, and mentioned in all relevant figure legends.

--------- Please don't hesitate to contact NCB@nature.com should you have queries about any of the above requirements ---------

Version 1:

Decision Letter:

Our ref: NCB-A60036A

27th March 2026

Dear Dr. Zanetti,

Thank you for submitting your revised manuscript "Multi-scale Molecular Imaging of Human Cells reveals COPI and COPII Vesicles at ER Exit Sites" (NCB-A60036A). It has now been seen by the original referees and their comments are below. The reviewers find that the paper has improved in revision, and therefore we'll be happy in principle to publish it in Nature Cell Biology, pending minor revisions to satisfy the referees' final requests and to comply with our editorial and formatting guidelines.

Please note that the current version of your manuscript is in a PDF format. Please email us a copy of the file in an editable format (Microsoft Word) as we cannot proceed with PDFs at this stage. Thank you in advance.

With the Word file in-hand, we will be performing detailed checks on your paper and will send you a checklist detailing our editorial and formatting requirements in about 1-2 weeks. Please do not upload the final materials and make any revisions until you receive this additional information from us.

Thank you again for your interest in Nature Cell Biology. Please do not hesitate to contact me if you have any questions.

Sincerely,

Melina Casadio, PhD
Senior Editor, Nature Cell Biology
ORCID ID: https://orcid.org/0000-0003-2389-2243

Reviewer #1 (Remarks to the Author):

I would like to thank the authors for being thorough in addressing comments. I believe my concerns have been addressed.

Reviewer #1 (Remarks on Protocol(s)):

The methods are well written and detailed.

Reviewer #2 (Remarks to the Author):

The authors addressed all my comments adequately, and the manuscript is now appropriate for publication. Congratulations on an excellent piece of work!

Reviewer #2 (Remarks on Protocol(s)):

I have reviewed the materials and methods section; no other additional protocol file. The materials and methods section, along with the supplementary figures, provides sufficient detail in my opinion and is written clearly.

Reviewer #3 (Remarks to the Author):

Downes et al. now present a significantly expanded and improved version of their manuscript “Multi-scale molecular imaging of human cells reveals COPI and COPII vesicles at ER exit sites.” In particular, the greatly expanded images in Figure 3 and the supplementary figures strengthen their point and now provide sufficiently compelling data to support their overall conclusions that COPI- and COPII- coated vesicles are present at ER exit sites in RPE-1 cells. Despite my excitement about this finding, I have to say I am still not convinced that they have the data to “clearly and unambiguously support of the vesicular trafficking model”, but I appreciate their attempts to address potential sources of artifact and provide more transparent methods section, so I am comfortable supporting the paper progressing towards publication despite my remaining concerns about overstated conclusions.

Reviewer #3 (Remarks on Protocol(s)):

The expanded paper and supplement provide sufficient details for me to understand the authors approach.

Version 2:

Decision Letter:

Dear Dr Zanetti,

I am pleased to inform you that your manuscript, "In situ cryo-ET defined the ultrastructure of ER exit sites in human cells", has now been accepted for publication in Nature Cell Biology.

Thank you for sending us the final manuscript files to be processed for print and online production, and for returning the manuscript checklists and other forms. Your manuscript will now be passed to our production team who will be in contact with you if there are any questions with the production quality of supplied figures and text.

Over the next few weeks, your paper will be copyedited to ensure that it conforms to Nature Cell Biology style. Once your paper is typeset, you will receive an email with a link to choose the appropriate publishing options for your paper and our Author Services team will be in touch regarding any additional information that may be required.

After the grant of rights is completed, you will receive a link to your electronic proof via email with a request to make any corrections within 48 hours. If, when you receive your proof, you cannot meet this deadline, please inform us at rjsproduction@springernature.com immediately.

You will not receive your proofs until the publishing agreement has been received through our system.

Due to the importance of these deadlines, we ask that you please let us know now whether you will be difficult to contact over the next month. If this is the case, we ask you provide us with the contact information (email, phone and fax) of someone who will be able to check the proofs on your behalf, and who will be available to address any last-minute problems.

If you have any questions about our publishing options, costs, Open Access requirements, or our legal forms, please contact ASJournals@springernature.com

Once your paper has been scheduled for online publication, the Nature press office will be in touch to confirm the details. An online order form for reprints of your paper is available at https://www.nature.com/reprints/author-reprints.html. All co-authors, authors' institutions and authors' funding agencies can order reprints using the form appropriate to their geographical region.

Publication is conditional on the manuscript not being published elsewhere and on there being no announcement of this work to any media outlet until the online publication date in Nature Cell Biology.

You may wish to make your media relations office aware of your accepted publication, in case they consider it appropriate to organize some internal or external publicity. Once your paper has been scheduled you will receive an email confirming the publication details. This is normally 3-4 working days in advance of publication. If you need additional notice of the date and time of publication, please let the production team know when you receive the proof of your article to ensure there is sufficient time to coordinate. Further information on our embargo policies can be found here: https://www.nature.com/authors/policies/embargo.html

Please note that *Nature Cell Biology* is a Transformative Journal (TJ). Authors may publish their research with us through the traditional subscription access route or make their paper immediately open access through payment of an article-processing charge (APC). Authors will not be required to make a final decision about access to their article until it has been accepted. Find out more about Transformative Journals

Authors may need to take specific actions to achieve compliance with funder and institutional open access mandates. If your research is supported by a funder that requires immediate open access (e.g. according to Plan S principles or the NIH public access policy) then you should select the gold OA route, and we will direct you to the compliant route where possible. Because authors warrant under our subscription licensing terms that they haven't committed to licensing any version of their article under a licence inconsistent with the terms of our agreement – including the applicable embargo period – publication under the subscription model isn't suitable for authors whose funders require no embargo.

To assist our authors in disseminating their research to the broader community, our SharedIt initiative provides you with a unique shareable link that will allow anyone (with or without a subscription) to read the published article. Recipients of the link with a subscription will also be able to download and print the PDF.

If your paper includes color figures, please be aware that in order to help cover some of the additional cost of four-color reproduction, Nature Portfolio charges our authors a fee for the printing of their color figures. Please contact our offices for exact pricing and details.

As soon as your article is published, you will receive an automated email with your shareable link.

If you have not already done so, we strongly recommend that you upload the step-by-step protocols used in this manuscript to protocols.io (https://protocols.io), an open online resource that allows researchers to share their detailed experimental know-how. All uploaded protocols are made freely available and are assigned DOIs for ease of citation. Protocols and Nature Portfolio journal papers in which they are used can be linked to one another, and this link is clearly and prominently visible in the online versions of both. Authors who performed the specific experiments can act as primary authors for the Protocol as they will be best placed to share the methodology details, but the Corresponding Author of the present research paper should be included as one of the authors. By uploading your Protocols onto protocols.io, you are enabling researchers

to more readily reproduce or adapt the methodology you use, as well as increasing the visibility of your protocols and papers. You can also establish a dedicated workspace to collect your lab Protocols. Further information can be found at https://www.protocols.io/help/publish-articles.

You can use a single sign-on for all your accounts, view the status of all your manuscript submissions and reviews, access usage statistics for your published articles and download a record of your refereeing activity for the Nature Portfolio.

Nature Cell Biology encourages authors presenting evidence for cell, biological, molecular, and genetic interactions to consider communicating these findings using Biofactoid (https://biofactoid.org/). This tool helps users share a searchable representation of interactions (e.g. binding, gene expression, post-translational modification) between genes, gene products, or chemicals. Information added to Biofactoid, with author attribution, is shared on social media and public databases, such as Pathway Commons, where it can be discovered and analyzed in the context of a large and growing corpus of knowledge.

Please feel free to contact us if you have any questions.

With kind regards,

Melina Casadio, PhD
Senior Editor, Nature Cell Biology
ORCID ID: https://orcid.org/0000-0003-2389-2243

Click here if you would like to recommend Nature Cell Biology to your librarian
http://www.nature.com/subscriptions/recommend.html#forms

** Visit the Springer Nature Editorial and Publishing website at www.springernature.com/editorial-and-publishing-jobs for more information about our career opportunities. If you have any questions please click here.**

Open Access This Peer Review File is licensed under a Creative Commons Attribution 4.0 International License, which permits use, sharing, adaptation, distribution and reproduction in any medium or format, as long as you give appropriate credit to the original author(s) and the source, provide a link to the Creative Commons license, and indicate if changes were made.
In cases where reviewers are anonymous, credit should be given to 'Anonymous Referee' and the source.
The images or other third party material in this Peer Review File are included in the article's Creative Commons license, unless indicated otherwise in a credit line to the material. If material is not included in the article's Creative Commons license and your intended use is not permitted by statutory regulation or exceeds the permitted use, you will need to obtain permission directly from the copyright holder.
To view a copy of this license, visit https://creativecommons.org/licenses/by/4.0/

We thank the reviewers for carefully evaluating the manuscript and providing insightful comments. We have addressed the reviewer comments to the best of our abilities, and we believe the revised version of the manuscript has significantly improved. A point by point response is below in green. Significant changes are highlighted within the manuscript in cyan.

Reviewer #1 (Remarks to the Author):

Summary
Downes et al. present an overview of COPI and COPII trafficking using cryo-electron tomography to unpick the arrangement, organisation and interaction of vesicles between ER exit sites (ERES) and the ER golgi intermediate compartment (ERGIC), as well as the wider context of Golgi. The authors present beautiful tomographic datasets and segmentations that allow the network of COPII and COPI vesicles to be seen in and amongst adjacent membrane compartments. The use of Halo Tags and Ligands in the context of live cell cryo electron tomography allows a wide range of questions to be addressed and presented here represents a simple elegant model to target ERES and ERGIC dynamics.
The authors use a low magnification (EM) step to help with fluorescence targeting which is technically novel and enriches the high magnification interpretation by widening the cellular observation field. While the identification and determination of size and shape the population of COPI and COPII vesicles is well established, the presentation of the data at time makes it hard to interpret how these sit into the wider context of the cellular tomograms.
Attempts are made to show this (Fig. 3), but the difficulty in interpreting the figures makes the results hard to follow. While there is enough data to be able to understand that what they conclude is, based on probability, correct, an edit of the figures to better annotate and illustrate the complex 3D structures that are present within the data and their relevance to the overall ERES-ERGIC COPI/COPII trafficking would be very beneficial.
The manuscript at time fails to present quantitative data to back up statements, of which I have made comment below. Furthermore, in some sections of the result the descriptions of the EM are too technical for a journal such as Nature Cell Biology and are in some cases irrelevant and should form part of the methods section.
Overall, the manuscript presents a wonderful leap in potential in understanding ER-Golgi interaction dynamics on the molecular scale, and with some edits and further quantification would be a good addition to the Nature Cell Biology portfolio.

Comments:
Line 102 and 105 – “unambiguously”.

We removed the first instance of “unambiguously”.

Lines 109 – 130 can be reduced in size, with some moved to the methods. I would condense this to focus on need to utilise a fluorescent model to increase the likelihood of encountering ERES targets and that they assessed the RPE-1 cell lines for its

functionality and ability to (Figure 1 and S1). It should also be made clear that the cryoET approach requires one that is live-cell compatible.

We have shortened this section, moving some details of the cell line validation to the methods, while others are explained in the legend of Supplementary Fig. 1. We modified as follows: “To improve targeting of ERESs in a live-cell compatible manner, we adopted a cryo-CLEM approach” to clarify why we chose a genetic fusion tag.

Lines 131-149. This paragraph focuses too much on the technical details of the EM and correlation. The choice of pixel size referenced is based on the ambition to perform structural studies on the COP vesicles but is not realised. Therefore, this context is largely superfluous. The authors aim to build the difficulties in targeting accurately with fluorescence into their adoption of an intermediate magnification tilt series, which is of technical note however for the purposes of the scope of the paper, which is very much cell biology focused, the merits of the technical parts here are less relevant. I would greatly reduce this section to focus solely on how ERES sites were achieved from the context of the cryoET structural cell biology and mentioned a short phrase to state that fluorescence combined with a low mag tilt series was the key aspect that allowed targeting of sufficient examples for further analysis.

The reviewer is right that since we have not done subtomogram averaging it seems superfluous to provide lengthy explanations about the choice for pixel size. However, we find that the intermediate magnification, high defocus step is necessary for targeting regardless of the magnification used for ‘final’ tomogram acquisition.
For this reason, our workflow is generally applicable to cell biology purposes. We have nevertheless shortened this section and moved most of the technical workflow details in the methods section, and removed the statements about aiming to do subtomogram averaging.

Line 141 – “restricted tilt series” what does “restricted” mean here?

It means with a tilt range restricted to +/-30 degrees and increment of 5 degrees. This is now moved to the methods.

Line 145 – 168. I do not see any reason for this to be included in the results here as it is mostly a methodological note with minimal relevance to the rest of what is written.
The portion on ribosomal structure determination should be written to reflect the ambition to work out ribosome-excluded regions of the data and quantitative measures of ERES localisation.

We modified this section as the reviewer suggests: we now start stating the aim to define ribosome exclusion zones (which we quantitate later as a response to the next comment), and we mention the tilt series improvement as an additional ‘bonus’ of this approach. We moved the part about handedness validation to the methods.

Line 201 – “Quantitative”. I find that in this section there is very little quantitation. It would have been useful to quantitate the ribosome exclusion zone, especially with the

extensive segmentation and particle position determination. Furthermore, how was the boundary delineated? A more thorough description/analysis would have provided insight into the global segregation of ERESs from the rest of the cytosolic constituents in a way that is less accessible to other techniques such as fluorescence.

We have now quantified the ribosome exclusion zone as follows: we used ArtiaX 'boundary' model on ribosome coordinates with an alpha value of 0.7 to delineate a tight boundary (figure below, middle panels) defining a ribosome 'inclusion zone'. We then created another boundary model using alpha = 1, which loosely encompasses all ribosomes (figure below, right panels). We calculated masks from both boundaries and subtracted the volume of the first from the volume of the second to generate the volume of the ribosome exclusion zone. This allows us to obtain an approximate value for the volume of the exclusion zone (the volume will be overestimated due to the indents around the surface in the 0.7 boundary mask, and underestimated due to the fact that the exclusion zone might be thicker than the lamella. This method works well unless there are big objects apart from ERES vesicles that exclude ribosomes (e.g. panel C), or unless the ERES vesicles are at the margin of the tomogram (panel D). We extrapolated an average diameter of the ribosome exclusion zone from 20 tomograms.

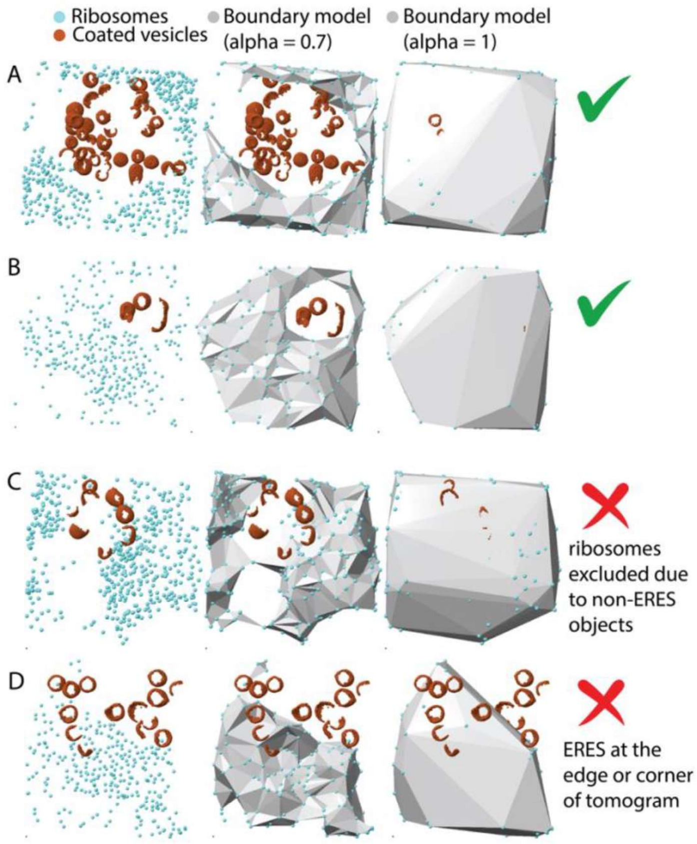

In addition, we measured the volume (and extrapolated average diameter) of the region encompassing coated vesicles by fitting a tight ellipsoid around them (Supplementary Fig. 4a). When we compare the two measurements we find the ribosome exclusion

zone is consistently ~200 nm larger than the diameter encompassed by coated vesicles. We now report and comment on this (Supplementary Fig. 4d).
We further quantified the ribosome density within these two regions and found that on average ERES regions have 10 fold less ribosomes than other cytosolic regions (Supplementary Fig. 4e).

Line 206 – "rather a sharp cut off" what does this phrase mean?

We removed the phrase.

Figure 3. This figure is very hard to interpret and is referenced in many places as an example of, often, quite intricate discussion on morphology and COPI, COPII and other phenomena. There is no annotation of the figure to illustrate points made in the manuscript and the caption is not helpful. Relevant places where figure 3 is referenced are ribosome density (line 206), vesicular-tubular clusters (lines 227-8), non-coated structures (line 247), buds (line 257), and vesicle morphology (line 381). Displaying whole tomogram segmentations of 9 tomograms, even with 3 colours to delineate COPII, COP1 and ribosomes, is a very difficult way to represent such a diverse range of important structure relevant to the core of this paper. I implore the authors to rethink this figure to tease out (annotate, use of insets, panels etc) details as well as showing the global organisation. I simply got lost trying to investigate thing reference in the text.

We have made a new version of this figure. We now show only two tomograms, including their 3D overview, and a number of insets to focus on COPII and COPI buds, vesicles, vesicular-tubular clusters with COPI. We include cytoskeleton segmentation for one of the tomograms, as requested by this reviewer at a later point. We made a separate figure to focus on ribosome density, to go alongside the expanded analysis of ribosome exclusion zones as described above (Supplementary Fig. 4). We keep the reference to uncoated and partially coated structures but have now included annotated details in Fig. 2. We also add views of the membrane segmentation along z, including a comparison with an ER tubule, to clarify the vesicular rather than tubular nature of coated structures, in response to Reviewer 3.
To give the reader an idea of the variations and similarities across tomograms, we decided to keep the original version with 9 different tomograms as Supplementary Fig. 7.

Line 211-212. What is high resolution confocal fluorescence microscopy?

To conduct the imaging studies described, we utilized the Nikon AX R with NSPARC microscopy system. The NSPARC detector allows for improved lateral resolution and signal to noise ratio. We also used a TIRF objective, which allows more light to be collected and thus better resolution. We used the wording, "high resolution microscopy" to highlight that our resolution obtained is greater than what is traditionally obtained using confocal microscopy but does not quite reach what is associated with the terminology "super resolution."
To avoid confusion, we have added more description to the text in the methods and

removed the use of “high resolution confocal” throughout the text, instead only referring to it as confocal microscopy.

Line 217-219. Is this sentence not essentially saying the same thing twice? To what end?

Yes, it is, we removed the second part and it now reads simply: “COPI coated structures were found in close proximity to COPII coated membranes in 86% of the tomograms.”

Line 219 – “Of the latter” Delete.

Done.

Line 220 – Use of high resolution (again) should be removed.

Changed.

Line 221 – How does the distance measurement of COPI to Sec23A+ puncta compare to the measurements in tomograms?

In order to compare distances measured in fluorescence with our cryo-ET data, we now measured the distance between the centre of mass of all COPII and all COPI coated events in each tomogram (Fig. 4d and e). We obtained values that are compatible with those measured by fluorescence. We now report this in the paper.

Line 222 – 77% of COPI puncta are not associated with COPII, but where is the quantification of the data presented? I could not find it, though I have to assume you had to count totals to calculate percentage.

We now report colocalisation quantification in Fig. 4c.

Line 227-228 – It is hard to discern the observations made within figure 3.

We made a new Fig. 3, please see response above.

Line 229. “High resolution” is used again.

Changed

Line 229-230 – Some quantification of relative positions observed would help here. Later in the paragraph, the fluorescence data (also in lines 231-233) are used to justify what the origin of COPI vesicles is within the tomography data (lines 233-235). It would as a reader to understand what prevalence there is in the data e.g. are COPI [, COPII] and ERGIC-53 always co-localised, majority of time, or less?

We have now included an additional Figure as Supplementary Figure 8 showing the percentage colocalisation of the various combinations of markers against COPI, COPII and the ERGIC. We report these values as the percentage calculated per image, where each image contains between 1-2 cells in the field of view (black points), as well as the overall percentage obtained across all images (white points), thus allowing the reader to assess the prevalence of the various structures observed in our data.

Line 236-237 – For COPII there are only 2 examples with more than 7 vesicles so that range 1-19 seems a little misleading (one could think it ranges all the way from 1 to 19, even though examples with 8 to 17 are not in the data). So it may be worth caveating the oddities in the range.

We have inverted the sentence to emphasise the majority observations before pointing out the exceptions. It now reads: "Most ERES have between 1 and 3 COPII coated membranes, inclusive of buds and vesicles (Fig. 5), however we observed a few instances of ERES with up to 19 COPII events (Fig. 5a). The number of COPI events in the majority of ERES is also between 1 and 3, with exceptional cases of up to 21 (Fig. 5b).

Line 250. Use of black star. I think it would be much more useful to excise this example as an inset and then 3D render the decoration with COPII across the vesicle surface to illustrate its "uncoating". As is, it is small and unannotated. Alternatively, approaches such as surforama may allow a world map of distribution to be rendered (10.1101/2024.01.05.574336)

We have generated insets for two partially coated vesicles in the new version of Fig. 2 and annotated them to illustrate the coated and uncoated portions. We attempted to generate figures with surforama, but this didn't work. Unfortunately, the automatic membrane segmentation does not distinguish between coated and uncoated membranes, and as a consequence, the segmentation mask is not 'flush' with the membrane, but rather it includes the coat where that is present. For this reason, surforama does not generate convincing differences between coated and uncoated regions.

Line 252. What do the authors mean by "anisotropy of the reconstruction"?

We intend the effect of the missing wedge, which 'wipes out' membrane and coat features as they approach an orientation perpendicular to the view axis. We modified the sentence to: "and the anisotropy of the reconstruction that weakens features along the view axis."

Line 257-258 – Where are the data to back up this statement?

For both COPI and COPII, there were either independent vesicles, or buds attached to uncoated 'donor' membranes. We are able to assign the nature of the donor compartments based on various morphological features: if continuous with ribosome-studded membranes, we assume it's ER (now shown in Fig. 3a,d). If arranged in stacked

and/or fenestrated cisternae, we assign to Golgi (Supplementary Fig. 9). When COPI buds were associated with smaller vesicular-tubular clusters, we have assigned these structures to ERGIC. All COPII buds we saw originated from the ER membrane, while for COPI it was either Golgi or ERGIC. We now show annotated examples of this in the new version of Fig. 3, but the full data is available deposited in EMPIAR database (EMPIAR-13270).

Line 268 - I could not see any quatification of the root mean square error related to the fit in Figure 5A.

As described in the methods, we also manually assigned the best fit (i.e. ellipsoid or sphere) to every structure upon visual inspection as we found this allowed the most appropriate fit to be assigned to each structure. To simplify this section, we have removed mention of the RMSE as we deemed this superfluous.

Line 269. For the volume calculations, how did you account for the missing wedge?

We used an iterative fitting algorithm to fit ellipsoid and spheres to incomplete shapes, following an approach used in Bieber et al [1]. Following this reviewer's comment, we analysed whether this approach is susceptible to missing wedge bias and we found that indeed it is: the azimuthal angle of the fitted ellipsoids longest axis is not random but skewed towards 90 degrees. However, while this affects our ability to determine sphericity (see response to point below from Reviewer 2 about sphericity), the volume measurements are not significantly affected, given most ellipses are only slightly elongated. To confirm this, we repeated measurements on 2D projections, and the distribution of volumes extrapolated from the average 2D diameter is undistinguishable from that measured in 3D.

Line 270. You give diameters based off the median volume, but how does the diameter compare to measurements from vesicles within the data? This would be good validation.

As outlined in the response above, we have now repeated the measurements on axial 2D projections of segmented vesicles and obtained size distributions that are indistinguishable from measurements made in 3D.

Line 278-279. As examples from tomography data are limited to Chlamydomonas and the mouse work is reconstituted (and therefore act as a contributory factor), use of the word species may be a bit strong and should be replaced to something along the line of phyla or kingdom (animals vs plants).

We substituted 'between species' with 'across evolution'.

Line 281-282. As there are ~ 4 points responsible for the skew, it would be useful to have the STDEV and ERRORs in the plot.

STDEV and standard error describe normally distributed data, as they are derived from the mean. These data on vesicle volume are not normally distributed, therefore presenting median and interquartile ranges is more appropriate. We now include these values in the Supplementary Fig. 9 legend. We also like to point out that interquartile ranges for COPI have lower values than COPII, despite the 4 outliers the reviewer refers to, indicating these outliers do not affect the conclusions.

How much does the 'restriction' of the low mag tilt series affect the interpretation of the organisation of the ERGIC, ERES and Golgi? If the tilt series is only at low angles, does this produce difficulties in segmenting and analysing in 3D?

We have not done any 3D segmentation of the low mag tomograms. The 3D reconstructions are very useful in separating overlapping small features (such as vesicles) along their projection directions but, as the reviewer points out, the information in the vertical direction is very limited due to the wide missing wedge. We therefore interpret and analyse the low mag tomograms based on slices through their XY plane.

Line 339. "Extending to 1.5nm resolution" Is this based from the ribosome reconstruction rather than the CTF fit?

Indeed, the reviewer is correct. We can safely assume that on average the tomograms contain information to at least 15A resolution based on the ribosome subtomogram average. This of course does not mean we can interpret them to that level, but we can use that number to state that biological molecules are preserved/undamaged to at least 15A. We have not used the CTF fit as the CTF signal wouldn't be affected by damage, potentially over-estimating the 'real' information content, given the potential effects of low magnification pre-exposures of 10 electrons.

Why was no comparison done for the TFG localisation with COPI?

Given previous work on TFG and its role in clustering COPII vesicles, our original aim was to further characterize the spatial relationship between TFG and COPII. As seen in previous published work and in the confocal imaging presented in Fig. 7A, the distance between TFG and Sec23A cannot be resolved using these systems and thus requires more powerful microscopy.
The spatial relationship between Sec23A and COPI on the other hand can be resolved using the AXR with NSPARC system, where we found an average distance of approximately 134 nm (Fig. 4b). Given that TFG and Sec23A are completely co-located when viewed by AX R microscopy it can be assumed that the distance between TFG and COPI is comparable to that of Sec23 and COPI.

Line 346-348 – Where are the data shown for resolving such tubules etc?

We have included one example of this in the new version of Fig. 3, also in response to comments from Reviewer 3.

Line 371-373 – As cytoskeleton is discussed, it may be worth having representative images of what is discussed from the data (that filaments are not interacting with the ER-Golgi network).

We have included a representative tomogram with segmented intermediate filaments and microtubules in the new version of Fig. 3. It is hard to show lack of interaction from a 2D image, but we added this tomogram to one of the representative tomograms to be deposited in EMDB (EMD-56606 and EMD-56951).

Figures:
Figure 1. The use of a star in 1D should be referenced in the caption. As mentioned above, to help with understanding uncoating it would be helpful to tease this out in a separate figure or panel. If in this figure, a row or vesicle types as panel E at the bottom may be one way to show this.

We removed the star, as we now focus on partially coated vesicle examples in Fig. 2.

Figure 2. There are 28 examples each of COPI and COPII. While it is interesting to see the diversity, I think it may be more worthwhile focusing on a subset here and then annotating the specifics in the difference in lattice organisation of COPI and COPII. The diversity may then potentially be shown as a supplement.

We modified Fig. 2 to show only a few examples of buds and vesicles, with larger panels and better annotations. We also included partially coated COPII vesicles and one uncoated vesicle for comparison. We have moved the complete gallery of vesicles to the supplementary Figures (Supplementary Fig. 5)

Figure 3. This needs to be properly reworked and annotated. The caption does not help. This figure is referenced four times in the text to illustrate several phenomena, and this could be presented appropriately to help the reader track what the different places in the text mean.

See response above, we believe we have now significantly improved this.

Supplementary Figure 4
As a further row, an enlarged, to-scale shot of the vesicle should be shown to contextualise the maps D-F.

We have modified this figure as requested (Now Supplementary Fig. 6)

Supplementary Figure 5
The ellipsoidal fit is not clear in A, and there is no quantification shown to back up the statement on line 268.

We have updated this panel to better show the fits which is now shown in Supplementary Fig. 9b. We have also further clarified the pipeline for this analysis in the

methods and removed reference to RMSE.

Reviewer #2 (Remarks to the Author):

This manuscript presents a thorough high-resolution description of the ER-ERGIC interface in human cells, with a focus on the arrangement of COPI and COPII at ER-exit sites. The authors use cryo-electron tomography combined with fluorescence microscopy. The results show very convincingly the occurrence of COPII coated vesicular carriers, a topic of recent debate. Particularly for this reason, but also for the large amount of other architectural details that lead to conclusions about dynamics, structure and mechanism, this study is of high interest for the cell biology and the membrane trafficking community. I therefore recommend publication of this manuscript, with some suggestions for revisions as follows.

Major:

1. Line 203 and following: It would be great to give some quantification of the percentage or number of vesicles vs. membrane buds already here, as it remains unclear how relevant the ultrastructure description is without numbers.

We added an introduction sentence that reads: "We found a total of 157 COPII coated membranes (95 vesicles and 62 buds), and 187 COPI coated membranes (39 vesicles and 148 buds). Coated membranes appear clustered…"

2. Line 216: Why was is not possible to quantify the displacement between ER and COPII vesicles from the cryo-ET data, to have a direct comparison with the confocal imaging data?

To perform such quantification in a meaningful way, we would need to precisely mark the position of transitional ER in our tomograms. We are not confident how to do this. In the fluorescence data, we use Sec16 as a proxy for the transitional ER, but we do not know where Sec16 is in the tomograms. There are often more than one COPII budding sites captured within a tomogram, and likewise there could be sites of Sec16 that haven't yet recruited visible amounts of COPII coat. When we said 'consistent with the cryo-ET data, we meant that the overall size of ERES-occupied areas (now quantified with ribosome exclusion zones, see above) are in the same range as the distances we observe in the fluorescence images. However, we realise this sentence can be confusing and we have now removed it.

3. Line 194-195: The statement "This suggests that the coat architecture described previously at high resolution is likely to be physiologically relevant" seems an overstatement. I agree with everything else concluded in this paragraph, but the here presented data does not allow to conclude that the high-resolution structures were correct (even if they likely were).

We removed this sentence

4. Line 257-8: How can the authors be so certain in all cases about the assignment of ER, ERGIC and Golgi membranes? In particular as in lines 242-3 they say it's difficult to distinguish ERGIC and Golgi.

The reviewer makes an important point here. ER and Golgi structures are clearly identifiable based on the presence of membranes studded with ribosomes or the arrangement in flat and often fenestrated cisternae (see also response to reviewer 1 and new version of Fig. 3). The lumenal density also appears different between ER and Golgi, with the Golgi being smoother and having a darker lumen. We call 'ERGIC' small vesicular-tubular clusters decorated with COPI buds, based on two observations: 1. In our STED images ERGIC-53 is surrounded by COPI and, 2. We also find these vesicular tubular clusters at a distance from the Golgi, suggesting they are distinct. This said, COPI also buds from the Golgi and where VTCs are in the Golgi vicinity we can't be sure of the identity of the membranes. This is why we say in the lines referred to here: "COPI buds were always associated with ERGIC or Golgi membranes". We do wonder whether it is semantically correct to attribute them to one or the other given the popular model where ERGIC matures to become a cis-Golgi cisterna. We have tried to make this point in the manuscript that reads as follows: "When ERESs are only a few hundred nanometers away from Golgi membranes, it can be hard to distinguish the ERGIC from the cis-Golgi cisterna. However, such distinction might be a matter of definition in a context where the ERGIC matures to become cis-Golgi."

5. Line 267: Could the apparent ellipsoidity of the vesicles be influenced by the anisotropic resolution / the missing wedge of the tomograms? How can this be excluded?

We would like to thank the reviewer for pointing this out, we have now checked for missing wedge bias and it is clear that there is bias, as the longest axis of the fitted ellipsoids are not randomly oriented. While this bias doesn't significantly affect volume measurements (see response to Reviewer 1 point above about volume measurement), it invalidates the sphericity analysis we reported in previous Fig. 5f. We have now removed this analysis.

6. Lines 294-297: How different is this analysis from the one of ellipsoid shape mentioned above, and why are they referred to in different parts of the manuscript?

It was in fact the same analysis. We now removed the description of sphericity (see point above).

7. The success rate of the CLEM protocol, especially the initial variant with directly acquiring high-magnification tomograms, seems very poor. Can the authors exclude that this is not only due to difficulties in alignments, but perhaps also due to false-positive signals, including accumulations of labelled Sec23 that is not assembled on membranes?

We would like to thank the reviewer for this comment as it led us to re-examine the data and establish that indeed our cryo-fluorescence images suffer from some degree of autofluorescence that maps to dense granules. This led us to conclude that some of the data we collected was targeted around fluorescent spots where the fluorescence was not coming from Sec23, which probably explains the low success rate. We have modified the text in the manuscript to reflect this. We also removed the fluorescence panels from Supplementary Fig. 2, as – whilst we were indeed guided by fluorescence – we now think this has not made a major impact in our correlative workflow. While we believe most of the false positives are associated with dense granules, we cannot exclude that there are false positive signals coming from Sec23 aggregates. However, Halo-Sec23 fluorescence at room temperature shows near-perfect co-localisation with Sec31 (Supplementary Fig. 1), so we think it is unlikely that puncta are Sec23 'aggregates', unless they form during the freezing process.

8. Along the same lines, in line 152/153 the authors that even with the optimised CLEM pipeline, 72% of regions of interest contained non-coated membranes. Is it possible to give an estimate of how certainly these did not contain Halo-labelled Sec23? And for the COPI containing tomograms mentioned in lines 223/224, how certain is it that these did not contain any Halo-labelled Sec23?

We cannot be certain that there is no Sec23 in these tomograms. All we can say is that there is not any Sec23 that polymerises into extended coats around membranes, as the latter are clearly visible. Small coat patches or individual 'soluble' Sec23 would be impossible to detect in noisy tomograms, even though they could still show fluorescent signal. However, as pointed out in the previous answer, we think the low success rate is largely attributable to autofluorescence.

9. Since the template matching for COPI and COPII worked well, why was the data not amenable to subtomogram averaging, as mentioned in the Discussion lines 384-385?

Probably due to low particle number: while coated vesicles show a radial density profile that is consistent with COPII and COPI (Fig. 2), averages from random oversampling of coated membranes are featureless and didn't work as starting references for subtomogram averaging. We chose not to perform subtomogram averaging using template-matched peaks as particles and/or previous structures as starting references as, given the number of particles and difficulty in aligning them, we would surely be trapped in template bias issues. This judgement comes from previous experience with aligning COPII coat subunits on in vitro reconstituted membranes on microsomes[2]: despite the purity, high SNR and high number of particles (see 'in vitro' panel of Fig. 2A), those particles were difficult to align and prone to template bias. Even within the best case scenario of in vitro samples, bias-free averaging only delivered an inner coat structure at ~15 Å resolution, as the COPII components assemble in small patches with high levels of heterogeneity. Overall, on balance, we deemed subtomogram averaging inappropriate.

Minor points:

10. Abstract: line 22: It is clear what “unperturbed” means. In fact, the cells are CRISPR edited, does that qualify as unperturbed? Or do the authors mean no drug treatment?

The cells are not treated with any drugs, temperature block, or overexpression system. We now spell this out to clarify.

11. Line 42-44: It would be good to back up this sentence with a reference.

Done

12. Line 84-85: I don’t seem to understand the sentence starting with “Live cell imaging…” e.g. what do you mean by steady state location?

We mean COPII puncta remain stable in ERES location over time. We now changed to ‘steady state at ERES’

13. In Figure 2, are the images showing in vitro vesicles shown at exactly the same scale as the others? If yes, please indicate in legend, if not, please make extra scale bars.

Yes, they are, we now indicate it in the legend

14. Please add numbers n to the legend of Figure 5 panels. In particular, panel D, E and F would benefit from indicating what n membranes or vesicles were analysed, and what n tomograms they came from (I know this info is in the text, but it’s difficult to access when looking at the figure)

Done

15. In the Results section, past and present tense are used inconsistently. For example, at about line 166 there is a sharp change to present tense.

We checked and corrected inconsistencies. We now use past to describe experiments and data, and present to provide interpretations.

16. In Supplementary Figure 8, I believe the label next to the purple vesicle should be COPI (not COPII).

Thanks for spotting this! We corrected it now (now Supplementary Fig. 12).

Reviewer #3 (Remarks to the Author):

Downes et al. present an exciting set of experiments using cryo-correlative fluorescence to address arguably the most contentious question today in secretory pathway biology: the nature of the ER exit site (ERES). They use a newly emerging tool in cryo-(high resolution template matching, HRTM) to find the structures of COPI and

COPII in lamella cut through putative ER exit sites, as identified using pre- and post-lamella cutting cryo-fluorescence from an endogenously-tagged RPE1 line. They further support this with some quantitative localization studies using STED microscopy to try to address the underlying reasons for some of what is observed in the cryoET data. This paper represents extremely laborious and expensive approaches, and I appreciate the significant effort the authors have applied to try solve one of the biggest questions in cell biology today.

In principle, this ought to be the landmark paper that finally puts the issue of ER exit site (ERES) structure to rest, as the only way I can even imagine this question being directly addressed is the approach presented here. However, the data as it is presented sort of drops the ball on this. I don't have any skin in this game, and even I am not clearly convinced by what is shown in the figures, despite some very aggressive phrasing on the part of the authors: "our results clearly and unambiguously support the vesicular transport model." If the authors really have the data to back this up, I will change my opinion, but as it is presented now this will be another in a long list of papers making big claims about mapping the mechanisms of ER exit without actually backing it up with their data. I have detailed my major and minor issues below.

The reviewer raises a series of concerns suggesting that our data are misinterpreted or artefactual. We address each of these points individually below. However, we also wish to note more generally that these arguments rely on a chain of speculative assumptions that, taken together, require multiple unlikely or unsupported scenarios to account for our observations. In contrast, a straightforward interpretation of the data - namely, the presence of COPII-coated vesicles at ER exit sites - provides a more parsimonious and experimentally supported explanation. While we fully acknowledge ongoing debates regarding alternative models of ER–Golgi transport, and do not dismiss them in our paper, we feel the reviewer's rejection of the vesicular interpretation is not warranted.

We also would like to point out that we did not use HRTM to find the structures of COPI and COPII. We use standard 3D template matching (where templates are heavily low-pass filtered) to obtain an unbiased confirmation of coat identity.

Major concerns:

1. The authors claim several times in the text that they can unequivocally identify vesicles as opposed tubular structures. I am confused why they have never shown them? Every "vesicle" I can see easily in the figures is truncated on one side or the other, and I do not see how they could possibly know unequivocally that this is a vesicle rather than a tube, especially given that the both theoretical and the most recent works arguing for tubes already stated that they frequently see pearling structures, which would appear as a vesicle if truncated in this way (see PMIDs: 33852913, 14645071, for example).

We most definitely see vesicles and not tubules or pearled structures. We are sorry that we didn't make this clearer with figures. Also in response to Reviewer 1, we now present a new version of Fig. 3 where we make the vesicular nature of coated membranes clear (See in particular panels f,h and i). The reason why segmented membranes appear open is due to the inherent 'missing wedge' (anisotropy) in the reconstruction. If these

vesicles were vertically oriented tubules, or pearled tubules, we would clearly see this in our reconstructions, as indicated in the new Fig. 3i panel where we directly compare a COPII vesicle with an ER tubule. In our case, no other membranes appear either above or below vesicles, indicating that they are closed and independent and not tubular. This can be independently assessed by users from the raw data that has been deposited on EMPIAR (Dep 13270).

We would like to point out that, if coated tubules were present, we would expect them to be randomly oriented within ERES. If the reviewer's suggestion that we are mistaking tubules for vesicles due to truncation was correct, this would mean that 157 out of 157 of the COPII-coated structures we detect (some of which shown in the gallery in Fig. 2, and Supplementary Fig. 5) are all oriented vertically along the viewing direction, which we consider quite improbable. To quantify this, we fitted ellipses to axial 2D projections of segmented COPI and COPII vesicles and calculated their circularity as the ratio between the shortest and longest elliptical axis. Closed near-spherical vesicles will have values close to 1 while projections of randomly oriented tubes are expected to range from spherical to strongly elliptical. Both types of coated vesicles ranged from ~0.8 – 1 with a median of ~0.9 (Supplementary Fig. 9a) showing that they are close-to-spherical in shape.

2. In general, I applaud the use of the emerging technique of template matching, but it comes with a risk that must be carefully modulated. Namely, it biases towards seeing what is used as the bait. Since the authors have used in vitro-derived COPII structures on vesicles as their bait, this runs the risk of only finding COPII in regions where it approximates a spherical structure. This would explain why they see such an imbalance between COPI and COPII on budding vs vesicular structures, since the structure of the inner coat is more well-defined. The use of pattern matching on "un-coated structures" is not an acceptable control for this, the authors need to evaluate how "flat" a structure can be before they fail to resolve it and think of an appropriate control for a) where they could be missing COPII and b) how often it can erroneously match a coated bud (seems unlikely, but this should be checked). At the very least, this needs a more significant treatment on the method that can allow a reader to critically evaluate this potential limitation. I am concerned this has biased the result to give exactly what the authors expect to see, rather than a fair evaluation of the data.

We ran the control requested and used the COPII structure from spherical vesicles (used to search our in situ tomograms) as a template to search cryo-tomograms of in vitro reconstituted samples which contain COPII coated spheres and tubules (amongst non-coated membranes). The template was successful in finding COPII on all membrane morphologies present, as shown in the figure below, where regions of tubular or flat coated membranes are highlighted in orange boxes.

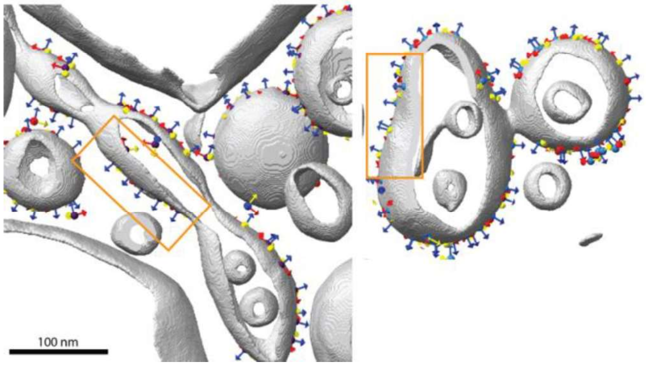

We also ran template matching to confirm our manual assignments (i.e. to discriminate coated membranes between COPI and COPII, rather than to 'find' coats).
The control we had performed, to run template matching using a COPII and a COPI template on identical sets of target tomograms is valid for the following reasons:

1. Both COPII and COPI structures used as templates are derived from the structure of coats on spherical vesicles, therefore there is no reason why COPII should 'prefer' spherical regions' over COPI (and anyway this is not what we see as both types of coated vesicles tend to be spherical).

2. Each template matching peak is independent of all others, and for the most part vesicles as well as buds are preferentially matched <u>either</u> by COPII only <u>or</u> by COPI only (Fig. 2). If the underlying data wasn't truly representing two different coats that resemble COPII and COPI respectively, then each coated membrane would be matched by a random collection of both templates, which is not what we see.

Our results therefore show that template matching is able to distinguish between the two coats.

3. I'm sorry to the authors because I know this is a hard critique to evaluate, but given the importance of this work and the boldness of the claims, the authors need to directly address the potential of vesiculation artifacts as a result of freezing. Plunge freezing is a challenging pipeline to execute for fragile membrane structures like this, if the cells have contacted the air for too long during blotting, it is well established in the field that thin tubules will vesiculate from transient osmotic stress.
Obviously, this has never been applied to exit sites, where we are not sure of the structure, but numerous people in the field have described this for thin peripheral ER tubules and sorting tubules on endosomes. The best way to verify that the freezing process was non-perturbative is to find an example of an endosome within one of the lamella and show that the tubules are intact and not fragmented, since the peripheral ER tubules will be hard to get in lamella and may respond differently to osmotic stress that things in the perinuclear region. Without some control of this type, I doubt the advocates for the lateral diffusion of proteins into the exit site will accept the results of the paper, and the controversy will continue.

We could not find any references to the stated phenomenon of "thin tubules vesiculating from transient osmotic stress" when freezing cells. In fact, we asked the international cryo-FIB/SEM community via a slack channel that hosts ~600 expert scientists and no one is aware of any data that shows this. We were made aware of one paper from Mageswaran et al in the Jensen lab[3] where plasma membrane is shown to shed vesicle-like protrusions when damaged (note this phenomenon doesn't occur in intact membranes). If this is the paper the reviewer is thinking of, then it is not relevant to our case as we see internal vesicles, not plasma membrane ones. If not, possibly the reviewer could point us to the data they refer to?

Internal cell membranes never come into contact with the air water interface during plunge freezing and are only 'exposed' upon milling in their frozen state, where such a change in conformation from tubules to vesicles is not possible. We wonder if the reviewer might have confused our cryo-FIB/SEM approach with in vitro systems where membranes are directly exposed to the air-water interface. Even considering this, we have previously imaged in vitro reconstituted COPII coated tubules[2,4,5], and those remain perfectly tubular upon contact with the air-water interface, so even if this 'artefactual vesiculation' phenomenon occurred on internal cell membranes (unlikely), it would not apply to COPII tubules.

As for the specific controls the reviewer suggests, we have no means to recognise endosomes in our data, as they are not known to have a coat. We see numerous instances of well-established uncoated ER tubules (and these are not vesiculating). We now show one such tubule in the new version of Fig. 3.

4. I am concerned by the lack of the authors to find microtubules in the vicinity of ERES, since those were so prevalent in the whole cell FIB-SEM volumes published. This begs one of two possibilities:

We regret the misunderstanding. As we stated in the paper we see microtubules in the vicinity of ERES in 72% of the tomograms. We now include cytoskeleton segmentation of one representative tomogram in Fig. 3g.

A) The lamella selection and validation is biasing the presented results towards ERES that do not have microtubules present. This is potentially a problem for the interpretations of the paper.

The number of microtubules is not significantly different (P=0.825) in ERES-containing and in ERES-free tomograms, as we clearly state in the paper, so this is a non-issue.

B) The nature of the lamella cutting is such that it nearly always removes the microtubule in proximity due to some inherent angling of the two with respect to one another and the angle of the FIB milling beam. This would be interesting, and is not a problem for the paper's interpretations, per se. This should be addressed, if possible.

This is not physically possible, as microtubules are oriented in all directions, and in any case as we point out above, we do see microtubules, so again, a non-issue.

5. Throughout the paper, the authors interpret “budding” vesicles as though they have captured a stage in a dynamic process. The data presented is static, of course, I do not think the authors can make this kind of inference from it. I recognize that interpreting the data this way amplifies the perceived impact, but if the authors cannot find a way to support their interpretations with something about the dynamic processes at this spot, I think they cannot make such firm statements. Is it possible their existing STED or NSPARC experimental pipeline could help with this? Live imaging of the recovery from bleaching, etc. might help to address tis directly.

We used the term ‘budding’ as in ‘buds attached to the organelle membrane, rather than to convey a dynamic process, we agree this terminology can be confusing and we changed ‘budding’ to ‘coated membrane buds’. Given that COPII buds are always attached to the ER and COPI to the ERGIC or Golgi (Examples in Fig. 3), we assume these are budding rather than fusion events. We make it clear in the paper that this is our inference, however we think it is a perfectly reasonable one due to the wealth of literature showing COPII mediates ER export and COPI mediates Golgi and ERGIC retrieval[6–10].

The buds we see are more likely to be snapshots of dynamic events, rather than static entities. Both COPII and COPI are known to be dynamically remodelling membranes. In our cells COPI is wild type and cells are untreated with any drugs or temperature block, so there is no reason to think that COPI dynamics are altered in our samples. As for COPII, the Halo tag on Sec23 is unlikely to affect COPII processes as overall dynamics of cargo export in WT and Halo-Sec23 cells are undistinguishable, as shown in the paper from the Audhya lab where the Halo-Sec23 cell line was originally introduced[11]. If there was an effect on coat dynamics from the Halo tag this would be reflected in overall cargo export.

STED and photobleaching experiments have been performed by many before us, and while very informative, they do not have the spatiotemporal resolution to assess the dynamics of individual coated membranes.

Minor Points:

1. The methods section is insufficiently detailed for me to critically evaluate some of the technical aspects of the paper. Some effort contributed to supplementary figures to illustrate the mechanics would go a long way in establishing trust in the approach. It would make it much easier if the code used to perfume the template matching, segmentation, and STED image analysis was provided.

We are not sure what the reviewer means by ‘illustrate the mechanics would go a long way in establishing trust in the approach’. We have provided all home-developed code (available on GitHub https://github.com/KTDownes/Multi-scale-Molecular-Imaging-of-Human-Cells-reveals-COPI-and-COPII-Vesicles-at-ER-Exit-Sites), otherwise when 3rd party software was used, we explained this in the methods. It would help us improve if the reviewer could point out exactly which technical aspects require more detail.

2. Are channel alignments performed for the STED imaging using fiducial markers? I can't find this explicitly written anywhere.

An Abberior STED system was used. This system does not require manual alignment and instead is equipped with an automatic alignment procedure. We now specify this in the methods.

3. Can the authors provide maps of where the lamella were cut in each cell in the supplement? For readers who frequently do EM, this would help to understand what membranes are visible in the tomograms. This could also potentially affect why the authors' results are potentially at odds with some published work, where the techniques used only work in the furthest edges of cells (e.g. 33852719). It's formally possible that both mechanisms are valid, but differentially applied based on subcellular location or polarity.

We are not aware such maps exist or are even possible to obtain. FIB/SEM creates a slice through a cell that therefore will encompass peripheral and central regions. However, it is not possible to map where cell centre and periphery will be on the lamella. However, we can make an assessment based on the intracellular features that we see in the TEM maps and low magnification tomograms. For example, where we see Golgi then we are more likely to be sampling perinuclear regions, where we see plasma membrane then we are more likely to be sampling peripheral regions. As we discuss in the paper, most ERES we see are likely perinuclear sites, but we did occasionally see peripheral sites too, and these appear similar in terms of their organisation (Supplementary Fig. 10). While it is possible that ERES organisation differs between perinuclear and peripheral sites, we have not seen this in our data, and further work targeting specific regions of the cell will be needed.

1. Bieber, A. *et al.* In situ structural analysis reveals membrane shape transitions during autophagosome formation. *Proceedings of the National Academy of Sciences* **119**, e2209823119 (2022).
2. Pyle, E., Miller, E. A. & Zanetti, G. Cryo-electron tomography reveals how COPII assembles on cargo-containing membranes. *Nat Struct Mol Biol* https://doi.org/10.1038/s41594-024-01413-4 (2024) doi:10.1038/s41594-024-01413-4.
3. A cryo–electron tomography workflow reveals protrusion-mediated shedding on injured plasma membrane | Science Advances. https://www.science.org/doi/full/10.1126/sciadv.abc6345.

4. Hutchings, J., Stancheva, V., Miller, E. A. & Zanetti, G. Subtomogram averaging of COPII assemblies reveals how coat organization dictates membrane shape. *Nature Communications* **9**, 4154 (2018).
5. Hutchings, J. *et al.* Structure of the complete, membrane-assembled COPII coat reveals a complex interaction network. *Nature Communications* **12**, 2034 (2021).
6. Barlowe, C. *et al.* COPII: a membrane coat formed by Sec proteins that drive vesicle budding from the endoplasmic reticulum. *Cell* **77**, 895–907 (1994).
7. Kuehn, M. J., Herrmann, J. M. & Schekman, R. COPII-cargo interactions direct protein sorting into ER-derived transport vesicles. *Nature* **391**, 187–190 (1998).
8. Novick, P., Ferro, S. & Schekman, R. Order of events in the yeast secretory pathway. *Cell* **25**, 461–469 (1981).
9. Arakel, E. C. & Schwappach, B. Formation of COPI-coated vesicles at a glance. *J Cell Sci* **131**, jcs209890 (2018).
10. Bykov, Y. S. *et al.* The structure of the COPI coat determined within the cell. *eLife* https://elifesciences.org/articles/32493 (2017) doi:10.7554/eLife.32493.
11. Kasberg, W., Luong, P., Swift, K. A. & Audhya, A. Nutrient deprivation alters the rate of COPII subunit recruitment at ER subdomains to tune secretory protein transport. *Nat Commun* **14**, 8140 (2023).
